# Frontotemporal Degeneration (FTD): Community Perspectives on Clinical Trials

**DOI:** 10.1002/alz.083598

**Published:** 2025-01-03

**Authors:** Shana G Dodge, Carrie Milliard, Penny A. Dacks

**Affiliations:** ^1^ The Association for Frontotemporal Degeneration (AFTD), King of Prussia, PA USA; ^2^ FTD Disorders Registry, King of Prussia, PA USA; ^3^ The Association for Frontotemporal Degeneration, King of Prussia, PA USA

## Abstract

**Background:**

Frontotemporal degeneration (FTD) is a complex, heterogeneous group of fatal adult‐onset disorders which lead to progressive dysfunction in behavior, motor symptoms, language, and/or cognition. While advances in research are cause for optimism, trials are hindered by the availability of participants. As FTD clinical trials typically require co‐participation of a study partner, care partner perspectives on research are critical to understanding how to support recruitment and retention.

**Method:**

An anonymized online survey queried aspects of the lived experience of FTD from the perspectives of people diagnosed, care partners, and biological family members from the United States, Canada, and the UK. The survey was circulated by the Association for Frontotemporal Degeneration (AFTD), the FTD Disorders Registry (FTDDR), and partner non‐profits, with 1800 complete responses, of which 1246 were from current or former care partners.

**Result:**

The percentage of care partners willing or very willing to participate in a clinical trial was highest amongst those caring for individuals at mild or moderate stages of disease (70% and 66%, respectively), though 49% of care partners were willing or very willing even at profound stages. Of those willing to participate, the diagnosed person’s reported willingness to answer detailed questions differed between those at earlier stages and those who were more progressed while lumbar punctures were the study procedure reported as least likely for the person diagnosed to be willing to undergo, at any stage of disease. Factors that increase willingness to participate included trial decentralization.

**Conclusion:**

FTD care partners are integral stakeholders in successful clinical trials. Their perceptions of clinical trials and openness to facilitating enrollment of the person diagnosed are moderated by factors such as disease severity and trial burden. Researchers must ensure the perspectives of people impacted by FTD are accounted for in trial design.

